# Genotyping and Descriptive Proteomics of a Potential Zoonotic Canine Strain of *Giardia duodenalis*, Infective to Mice

**DOI:** 10.1371/journal.pone.0164946

**Published:** 2016-10-19

**Authors:** Camila Henriques Coelho, Adriana Oliveira Costa, Ana Carolina Carvalho Silva, Maíra Mazzoni Pucci, Angela Vieira Serufo, Haendel Goncalves Nogueira Oliveira Busatti, Maurício Durigan, Jonas Perales, Alex Chapeaurouge, Daniel Almeida da Silva e Silva, Maria Aparecida Gomes, Juliano Simões Toledo, Steven M. Singer, Rosiane A. Silva-Pereira, Ana Paula Fernandes

**Affiliations:** 1 Departamento de Analises Clinicas e Toxicológicas – Faculdade de Farmácia, Universidade Federal de Minas Gerais, Belo Horizonte, Brazil; 2 Centro de Pesquisas René Rachou - FIOCRUZ/MG, Belo Horizonte, Minas Gerais, Brazil; 3 Centro de Biologia Molecular e Engenharia Genética (CBMEG-UNICAMP), Campinas, Brazil; 4 Laboratório de Toxinologia, Instituto Oswaldo Cruz- FIOCRUZ/RJ, Rio de Janeiro, Brazil; 5 Departamento de Biologia Geral, Universidade Federal de Minas Gerais, Belo Horizonte, Brazil; 6 Departamento de Parasitologia, Universidade Federal de Minas Gerais, Belo Horizonte, Brazil; 7 Biology Department – Georgetown University, Washington, United States of America; National Research Council, ITALY

## Abstract

The zoonotic potential of giardiasis, as proposed by WHO since the late 70's, has been largely confirmed in this century. The genetic assemblages A and B of *Giardia duodenalis* are frequently isolated from human and canine hosts. Most of the assemblage A strains are not infective to adult mice, which can limit the range of studies regarding to biology of *G*. *duodenalis*, including virulence factors and the interaction with host immune system. This study aimed to determine the infectivity in mice of an assemblage A *Giardia duodenalis* strain (BHFC1) isolated from a dog and to classify the strain in sub-assemblages (AI, AII, AIII) through the phylogenetic analysis of beta-giardin (bg), triose phosphate isomerase (*tpi*) and glutamate dehydrogenase (*gdh*) genes. In addition, the proteomic profile of soluble and insoluble protein fractions of trophozoites was analyzed by 2D-electrophoresis. Accordingly, trophozoites of BHFC1 were highly infective to Swiss mice. The phylogenetic analysis of *tpi* and *gdh* revealed that BHFC1 clustered to sub-assemblage AI. The proteomic map of soluble and insoluble protein fractions led to the identification of 187 proteins of *G*. *duodenalis*, 27 of them corresponding to hypothetical proteins. Considering both soluble and soluble fractions, the vast majority of the identified proteins (n = 82) were classified as metabolic proteins, mainly associated with carbon and lipid metabolism, including 53 proteins with catalytic activity. Some of the identified proteins correspond to antigens while others can be correlated with virulence. Besides a significant complementation to the proteomic data of *G*. *duodenalis*, these data provide an important source of information for future studies on various aspects of the biology of this parasite, such as virulence factors and host and pathogen interactions.

## Introduction

The enteric parasite *Giardia duodenalis* is the main protozoan causative agent of diarrhea, affecting millions of people worldwide [1, 2]. In developed countries, the prevalence ranges from 1% to 7.6%, while in developing countries it may reach 30% [3]. The majority of giardiasis reported cases occurs after transmission of *G*. *duodenalis* cysts through the ingestion of contaminated water and food. However, giardiasis may also be transmitted from domestic animal to man, as a zoonotic disease. [4].

The occurrence of eight genetic assemblages of *G*. *duodenalis* (A to H) has been depicted from molecular epidemiology analyses. Assemblages C and D are usually infective to dogs, while the assemblage A is considered zoonotic, infecting both humans and domestic animals, mainly cats and dogs [5, 6]. These zoonotic infections are usually associated to sub-assemblages AI and AII, which share high chromosome synteny and phylogeny. However, they can differ regarding their infectivity and pathogenic properties in mice models [7].

Proteomic analyses have revealed important biological traits in *G*. *duodenalis*, including those associated to virulence, encystation process and antigenic variation [8–12]. Moreover, through the generation of *G*. *duodenalis* proteomic datasets, new tools have become available for taxonomical analysis of this intestinal pathogen. Recently, the proteomes of eight *G*. *duodenalis* assemblage A strains (seven AI and one AII) isolated from human, cat, sheep and bird were obtained, allowing a more complete covering of the available data [13]. This analyss has disclosed specific sub-assemblage differences in protein identifications, especially for the variable antigens in the cell surface.

*G*. *duodenalis* canine isolates are not very frequently available and have been far less studied than human isolates, though infection in dogs is an epidemiological noteworthy aspect of *G*. *duodenalis* biology. Domestic dogs usually live in very close contact to humans and high rates of *G*. *duodenalis* infection in dogs have been reported in countries such as Brazil (36.8%), Italy (26.6%), Japan (23.4%), Belgium (22.7%) and UK (21.0%) [14–18], factors that potentially increase the transmission rates.

An isolate of *G*. *duodenalis* (BHFC1 strain) was previously obtained from dog stools, in Brazil. A preliminary genotyping of this strain revealed that it belongs to genotype A [19] and, therefore, could be a potential source of zoonotic transmission to humans. In the present work, this canine isolate of *G*. *duodenalis* was further characterized by phylogenetic analysis in order to determine its sub-assemblage (AI, AII or AIII). We have also evaluated its infectivity profile in Swiss mice and performed a proteomic mapping of BHFC1 trophozoites in both soluble and insoluble protein fractions.

## Material and Methods

### Parasites and axenization

The BHFC1 strain of *Giardia duodenalis*, previously isolated from dog stools in Belo Horizonte city, Brazil [19], was utilized in this study. Trophozoites were axenically cultivated in TYI-S-33 medium [20] at 36.5°C. The Portland-1 strain, cultivated under the same conditions, was used as control to confirm the genotyping procedures and to compare infectivity in mice.

### Mice infection

Female 6-week-old Swiss mice, were obtained from the animal facility of the Faculty of Pharmacy, Federal University of Minas Gerais (UFMG). Mice were kept under observation for 1 week prior to the experiment and were maintained under a 12-h light-dark cycle with *ad libitum* access to water and food. None of the animals became ill or died prior to the experimental endpoint. Mice were treated with Metronidazole and their stools were tested for giardiasis after lugol staining, in order to exclude previous infection with *Giardia muris*.

Swiss mice received 1x10^6^ trophozoites of BHFC1 in 0.2 mL of sterile phosphate-buffered saline (PBS), administrated through orogastric gavage. A control group received 1x10^6^ trophozoites of Portland-1, following the same procedure. This experiment was conducted in triplicate, with a minimal number of 3 mice per group (total of 10 mice, per strain) and mean and standard deviations were calculated for each group. The physical conditions of mice were monitored every 8 hours. After 7 days from inoculation, animals were anesthetized with 60 mg/Kg of ketamine and 8 mg/Kg of xylazine and were euthanized by cervical displacement. A 15 cm proximal segment of the small intestine (duodenum and jejunum) was removed and placed in 5 mL of sterile PBS. The tissue fragments were cut in 1 cm transversal sections and vortexed for 30s to release trophozoites from the intestinal wall. Trophozoites from the supernatant were quantified by counting in Neubauer chamber. All experiments were conducted in adherence to the animal protocol (7/2012) approved by the Ethics Committee on the use of Animals (CEUA) of the Federal University of Minas Gerais.

### Phylogenetic analysis

In order to determine in which *G*. *duodenalis* subassemblage (AI, AII or AIII) BHFC1 strain could be classified, nested PCR fragments of beta-giardin (*bg*), triose phosphate isomerase (*tpi*) and glutamate dehydrogenase (*gdh*) genes were sequenced. Genomic DNA from trophozoites was obtained by purification using the kit gDNA Charge Switch Mini Tissue^™^ (Invitrogen) and subjected to nested-PCR using the primers described in S1 Table. Nested PCR products were analyzed in 2% agarose gel and purified using Wizard Genomic DNA Purification^™^ kit (Promega, USA), according to the manufacturer's protocol. PCR products were sequenced by ABI3130 platform (Applied Biosystems, USA) at the company Myleus Biotecnology^™^.

Sequences obtained from the three loci were concatenated to define multilocus genotypes (MLGs). The Bayesian phylogenetic analysis was performed with MrBayes software version 3.1.2 [21] using the GTR model with gamma correction (the available model most similar to Tamura-Nei). The starting trees were random, Markov chains were run for 6,000,000 iterations, and the trees were sampled every 100 iterations. Bayesian posterior probabilities were calculated using a Markov chain Monte Carlo sampling technique. The Bayesian inferred trees were visualized with TreeView X [22]

Outgroups used for construction of phylogenetic trees were: *G*. *muris* for *bg* and *tpi* sequences, *Giardia ardeae* for *gdh* and *tpi*, and *G*. *microti* for *tpi* (S2 Table).The sequences generated in this study were deposited in GenBank under the following accession numbers: CCPORbg KT258017, CCBHbg KT258018, CCPORtpi KT258019, CCPORgdh KT258021, CCBHgdh KT258022.

### Protein extraction for proteomic analysis

Trophozoites collected after 72 hours of culture in TYI-S-33 medium were centrifuged (2,000 g, 10 minutes, 20°C) and washed three times with PBS (pH 7.2). The remaining sediment was suspended in 1.5 mL of PBS and transferred to a 2 mL tube and centrifuged per 5 minutes at 3,000g at 4°C. The proteins were extracted from trophozoites by the addition of 2DE lysis buffer (kit 2D Fractionation^™^, GE Healthcare), at a ratio of 200 μL for 1.4 x 10^9^ parasites. In order to obtain both soluble and insoluble protein fractions, the kit instructions were followed, with some adaptations. Originally, the manufacturer suggests up to 5 sub-fractions of the soluble portion of proteins. In contrast, only the first fraction of soluble proteins (Fraction I) and the insoluble fraction were obtained. Proteins were quantified by the Bradford method and stored at -70°C. After determination of protein concentrations, 1 μg of protein from each fraction (soluble or insoluble) was submitted to one-dimensional electrophoretic separation on 12% polyacrylamide gels, to assess the quality of the extracts and then to 2D gel electrophoresis procedure.

#### 2-D Electrophoresis

The protein extracts (500 μg) were solubilized in IEF rehydration buffer (8M urea, 2M thiourea, 4% CHAPS, 0.5% bromophenol blue, 65 μM DTT and 1% BioLyte 3–10 buffer 100X (Bio-Rad) to a final volume of 350 μL.

After stirring for one hour at room temperature, the samples were centrifuged at 16,000 g for 30 minutes at 25°C to remove non-solubilized material. The supernatant was loaded onto 17 cm IPG strips (Bio-Rad) of pH 3–10 non-linear gradient by in-gel sample rehydration and, after 10 minutes, 1.5 mL of mineral oil was overlaid onto strips. Isoelectric focusing was performed in the Protean IEF Cell (Bio-Rad) at 20°C and 50 μA/strip. Passive rehydration was carried out for 4 hours, followed by active rehydration at 50V, for 12 hours. Isoelectric focusing was performed, in gradient fashion, at 500V for one hour; 1,000V for 1 hour; 8,000V for 2 hours and from 8,000V until reach 40,000V/hour. After isoelectric focusing, the excess of mineral oil was removed and the strips were frozen at -70°C. In the second dimension, proteins were separated, in 12% SDS-PAGE gels.

Before SDS-PAGE, IPG strips were first maintained for 10 minutes in 5 mL of Equilibration Buffer I (50mM Tris-HCl, pH8.8, 6M urea, 30% glycerol, 2% SDS, 0.5% Bromophenol Blue and 130mMDTT) and, thereafter, for 10 minutes in Equilibration Buffer II (50mM Tris-HCl, pH8.8, 6M urea, 30% glycerol, 2% SDS, 0.5% Bromophenol Blue and 135mM Iodoacetamide). The molecular weight standard (Broad Range^™^, Bio-Rad) was applied on filter paper, placed on top of polyacrylamide gel and sealed with 0.5% agarose containing bromophenol blue. The IPG strips were washed in SDS-PAGE electrode buffer (25mM Tris, 192mM glycine, 0.1% SDS), and sealed with 0.5% agarose on top of 12% polyacrylamide gels.

Electrophoresis was carried out in a Protean II XL Multi-Cell^™^ (Bio-Rad) connected to a Multitemp II cooling bath (Amersham Biosciences) at 16°C under 50V, constant voltage, for the first hour and then under 200V, until the front dye reached the bottom of the gel. The gels were stained with Colloidal Coomassie Blue G-250 [23]. The 2D stained gels were scanned using a GS-800 densitometer (Bio-Rad), at a resolution of 300dpi, and then stored at 4°C, in 25% ammonium sulfate solution prior to perform excision of the spots for mass spectrometry. The images of duplicate 2D gels were analyzed by PDQuest^™^ 8.0.1 software (Bio-Rad).

#### MALDI-ToF Mass Spectrometry

Each spot of interest was located and manually excised from the gel for the mass spectrometry identification. The excised spots were washed twice for 15 minutes in 400 μL 50% acetonitrile (Fisher Scientific) and 25mM ammonium bicarbonate (Sigma), pH 8.0, until removal of the blue stain. Acetonitrile (200 μL) (Fisher Scientific—USA) was used to dehydrate the gel pieces. After dehydration acetonitrile was removed and the gels were dried in a Speed Vac Concentrator Plus^™^ (Eppendorf) for 15 minutes. Afterwards, 10 μL of 20 μg/mL Sequencing Grade Modified Trypsin (Promega) were added and 10 minutes after, 50 μL of 25 mM ammonium bicarbonate (Sigma), pH 8.0, were added. Tubes were incubated for 16 hours at 37°C, for protein digestion. The solution was transferred to a clean tube. To the tube containing pieces 30 μL of 5% formic acid (Merck—USA) and 50% acetonitrile (Fisher Scientific—USA) were added for extraction of tryptic peptides. This procedure was performed twice under stirring for 30 minutes each. The supernatant was pooled to the respective tube containing the initial peptide solution. The samples were concentrated in a SpeedVac to a volume of about 10 μL, and then the peptides were desalted in reversed phase micro columns Zip Tip C18 ^™^ (Eppendorf—Germany), according to the manufacturer's instructions.

Tandem mass spectra for protein identification were obtained on an AB Sciex 5800 (AB Sciex, Foster City, CA) MALDI mass spectrometer. Usually, up to twelve of the most intense peaks of each spot were selected for MS/MS acquisition, while masses related to trypsin autolysis products and common keratin masses were excluded. Calibration in MS mode was performed using a standard of six peptides including the following molecular masses: des-Arg1-Bradykinin (m/z = 904.468), angiotensin I (m/z = 1296.685), Glu1-fibrinopeptideB (m/z = 1570.677), ACTH (1–17 clip) (m/z = 2093.087), ACTH (18–39 clip) (m/z = 2465.199), and ACTH (7–38 clip) (m/z = 3,657.929). In addition, MS/MS spectra were calibrated by matching up to five fragment ions in the tandem mass spectrum of Glu1-fibrinopeptideB (m/z = 1570.677). The peak lists of these experiments were searched against an in-house created parasite database (117944 sequences) using the program Mascot (Mascot version 2.1). The search parameters included tryptic cleavage products, two tryptic missed cleavages allowed, and variable modifications of cysteine (carbamidomethylation), methionine (oxidation), asparagine and glutamine (deamidation), as well as pyroglutamate formation at N-terminal glutamine of peptides. To obtain maximum confidence in protein identification, p-values were finally adjusted to reach false discovery rates (FDR) of 1 percent or below.

The proteomic data was analyzed against the GiardiaDB platform (www.giardiadb.org), in order to evaluate protein features and the presence of signal peptide and membrane domains. In order to find the major biological functions of the identified proteins, graphics were constructed using the software Panther^®^, according to their Gene Ontology reference. All the proteins identified in this work were deposited in the Giardia DB.

## Results

### The BHFC1 strain belongs to sub-assemblage AI

**Fig 1:**
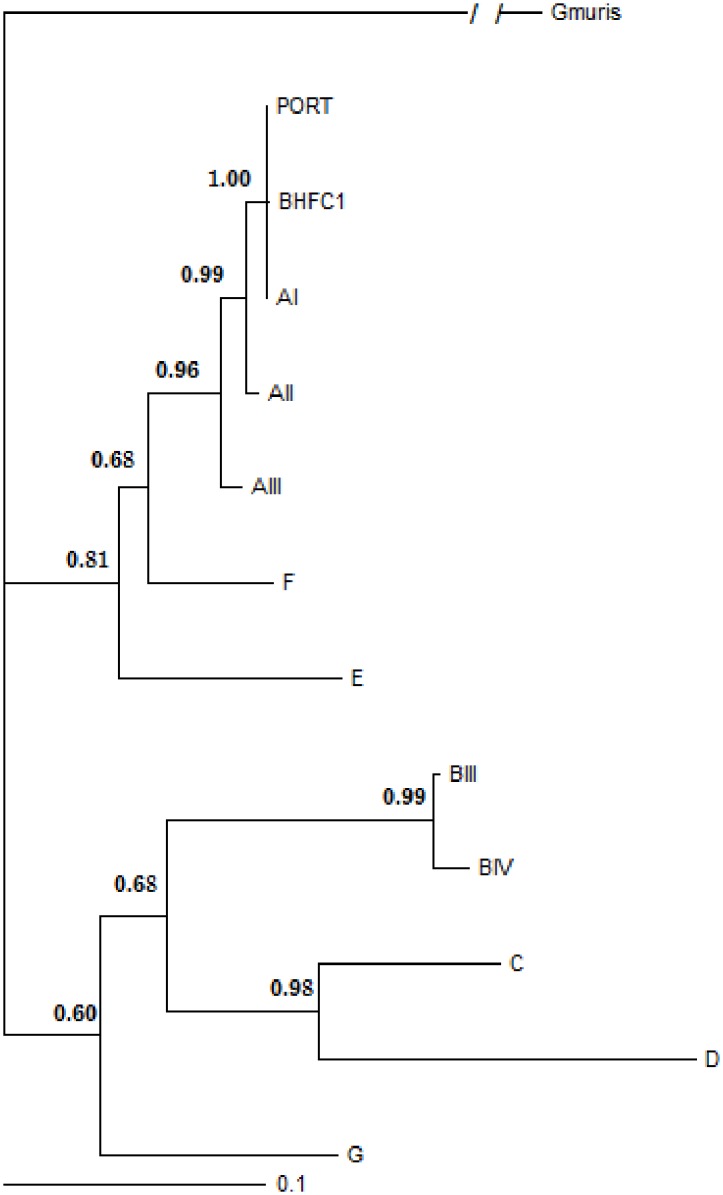
Consensus phylogenetic relationships of *G*. *duodenalis* with Bayesian posterior probabilities using a Markov chain Monte Carlo sampling technique, for *bg*, *tpi*, *and gdh* gene sequences of *G*. *duodenalis*. Markov chains were run for 6,000,000 iterations and the trees were sampled every 100 iterations. The GTR model was used with gamma correction. Sequences from *Giardia muris*, *Giardia microti and Giardia ardeae* were employed as outgroups.

In order to establish the phylogenetic relationships of the canine strain according to *G*. *duodenalis* subassemblages (AI to AIII), nested-PCR products obtained for the genes *bg*, *tpi* and *gdh* of BHFC1 and Portland-1 (control) were sequenced. The phylogenetic analysis discloses that BHFC1 shares a common ancestor with assemblage A strains. However, the phylogeny obtained with *tpi* and *gdh* genes sequences clustered BHFC1 to sub-assemblage AI, while *bg* analysis clustered it to AII assemblage. Then, a consensus phylogenetic analysis using *gdh*, *bg* and *tpi* genes with Bayesian posterior probabilities was performed (Fig 1), after alignment with additional public sequences of other assemblages (S2 Table). The canine strain was also clustered in the AI assemblage, according to this analysis, as well as the Portland-1 reference strain.

### The BHFC1 strain is infective to Swiss mice

The quantitative results obtained by counting of trophozoites or cysts recovered from the small intestine of Swiss mice after infection with BHFC1 and Portland-1, are shown in Table 1. While infection with BHFC1 strain resulted in recovering high numbers of trophozoites, but not cysts, in three independent experiments, only cysts, in low numbers, were recovered from mice infected with Portland-1.

**Table 1:** Number of trophozoites or cysts recovered from Swiss mice gut after infection with *G*. *duodenalis* strains BHCF1 and Portland-1. Mice were inoculated with 1 x 10^6^ trophozoites through intra-gastric route. Parasites were recovered from gut and counted in Neubauer chamber. Numbers correspond to average and standard deviation of parasite counting’s obtained for each group, from three independent experiments, with minimal number of 3 mice per group, in each experiment.

Strain	Mean and Standard Deviation of parasite counts		
	Experiment 1 (n = 3)	Experiment 2(n = 4)	Experiment 3 (n = 4)
**BHFC1**^a^	29,167 ± 21,262	135,000 ±112,110	121,667±98,752
**Portland-1**^b^	500 ± 866.02	333.33±577.35	0±0

^a^ Only trophozoites were recovered

^b^ Only cysts were recovered

### The proteomic map disclosed new hypothetical proteins of *G*. *duodenalis* and an active metabolic profile for BHFC1 canine strain

**Fig 2:**
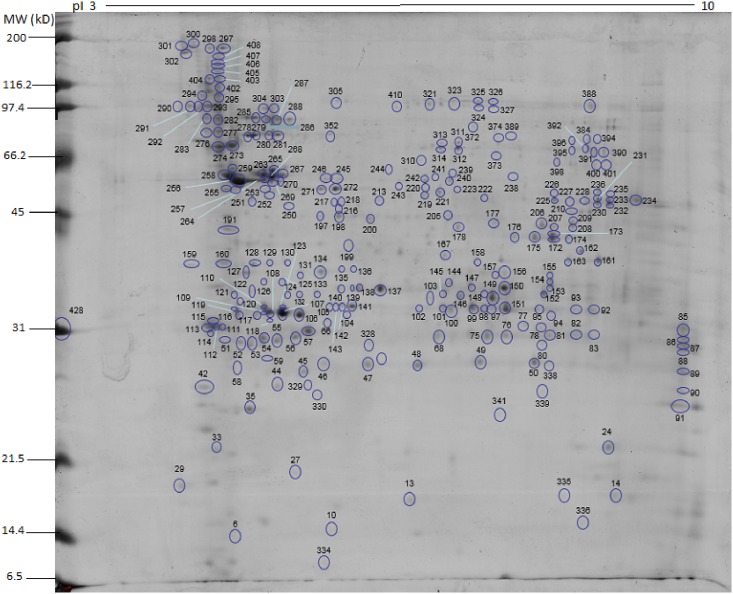
2D protein map showing the spots of the soluble protein fraction (Proteins 1 to 429) and the numerical distribution of localized proteins. The protein identification correspondent to each number is shown in S3 Table.

**Fig 3:**
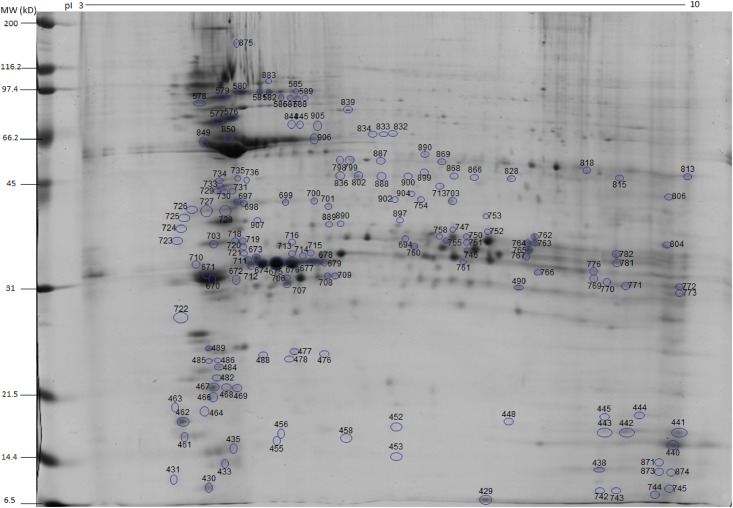
2D protein map showing the spots of the insoluble fraction (Proteins 430 to 903) and the numerical distribution of localized proteins. The protein identification correspondent to each number is shown in S3 Table.

Spots obtained either from soluble or insoluble protein extracts were localized in the 2D gels, and labeled with numbers 1 to 903 during the gel excision procedure. From the soluble protein fraction gel, 429 spots were excised, and from the insoluble protein fraction gel, 474 spots (Figs 2 and 3). The proteomic map was elaborated using the localization of each protein according to their migration in the first and second dimensions. After the mass spectrometry analysis, this proteomic map revealed 187 protein access numbers (S3 Table). Among these, 160 proteins had been previously annotated, whilst 27 were identified as hypothetical. Among the 160 proteins, 79 were identified in the insoluble protein fraction, 53 in the soluble protein fraction, and 28 presented peptides from both insoluble and insoluble protein fractions. Among the 27 hypothetical proteins, 20 were identified in the insoluble protein fraction, four in the soluble protein fraction, and 3 were detected in both insoluble and soluble protein fractions. Additional information related to the previously annotated hypothetical proteins is described in Table 2. Among the 187 proteins identified by mass spectrometry, 5 were related to other parasites and are described in the S4 Table.

**Table 2:** Characteristics of the identified hypothetical proteins, including the access number, the number of amino acids (aa) and the presence of putative conserved domains according to Protein Blast webtool (http://blast.ncbi.nlm.nih.gov).

ORF number	Number of amino acids	Conserved domains
GL50803_10016	317	Fascin Superfamily
GL50803_10524	250	Absent
GL50803_10808	228	Absent
GL50803_10524	837	Absent
GL50803_115159	644	Phospholipase B
GL50803_12224	300	Smc (Structural maintenance of chromosomes)
GL50803_13584	386	Smc
GL50803_21628	184	Absent
GL50803_15499	431	Sm (Archae type)
GL50803_15918	228	Absent
GL50803_16267	537	Trichoplein
GL50803_16424	252	Mlf1IP (Myelodysplasia-myeloid leukemia factor 1-interacting protein)
GL50803_16507	875	Absent
GL50803_16844	324	Absent
GL50803_16996	413	Enkurin (Calmodulin-binding)
GL50803_17278	297	Absent
GL50803_2107	328	Absent
GLC50803_21628	383	Absent
GL50803_24451	139	FGF family (Fibroblast growth factors)
GL50803_3910	123	TRX Family
GL50803_4149	440	Hom_end_hint
GL50803_4239	98	Absent
GL50803_4692	239	Absent
GL50803_5810	131	Pyridoxine 5'-phosphate(PNPOx-like)
GL50803_19861	385	Absent
GLP15_2507	821	Absent
GL50803_115159	644	Phospholipase B

**Fig 4:**
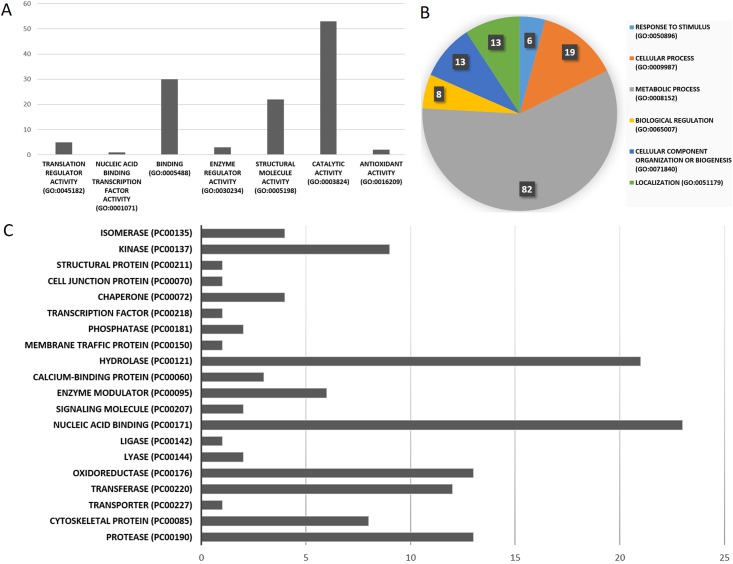
Classification of the identified proteins by molecular function (A), biological process (B) and protein class (C). This classification is suggested by gene ontology and Panther^®^ and provides a general characterization of a group of proteins. The numbers presented in each graph correspond to the number of proteins.

The identified proteins were further classified according to their molecular function, biological process and protein class (Fig 4). According to these classifications, the majority (57%) of the proteins were either involved in metabolic processes, including carbon metabolism or act on cellular processes, and 50 (31.2%) proteins have a catalytic activity. Nucleic acid binding proteins (14.4%), hydrolases (13.7%) and oxidoreductases (7.5%) were also highly prevalent (Fig 4c).

In order to evaluate whether the proteins identified potentially presented characteristics in the genetic annotation databases that could indicate its localization on plasmatic cell membrane, a search against the GiardiaDB was performed for presence of transmembrane domains and signal peptides in these proteins. This search resulted in 32 proteins presenting signal peptides. Of those, 26 were identified in the insoluble protein fraction. Eleven proteins presented transmembrane domains, seven of them from the insoluble fraction and four from the soluble. Among the ten proteins displaying both transmembrane domain and signal peptide, seven were present in the insoluble protein fraction (Table 3). Three proteins containing signal peptides were concomitantly present in soluble and insoluble protein fractions, which may be related to their intracellular trafficking.

**Table 3:** Presence of transmembrane domains and signal peptide for the annoted proteins identified in insoluble and soluble protein fractions.

Protein Description	ORF name	TD	SP	Insoluble fraction	Soluble fraction
Cathepsin L precursor	GL50803_16380		X	X	
Variant-Specific Surface Protein 160 (VSP-160)	GL50803_137612	X	X	X	
Cathepsin B precursor	GL50803_14019		X	X	
Tenascin-like	GL50803_16833		X	X	
Thymus-specific serine protease precursor	GL50803_10843		X	X	
Adenylate kinase	GL50803_90402		X	X	
PDI5—Protein disulfide isomerase	GL50803_8064	X	X		X
Variant-Specific Surface Protein 126.1 (VSP-126.1)	GL50803_11521		X	X	X
Leucine-rich repeat protein	GL50803_4039		X	X	X
Leucine-rich repeat protein 1 virus receptor protein	GL50803_5795		X	X	X
Variant-Specific Surface Protein 100 (VSP-100)	GL50803_33279	X	X	X	
Serine peptidase, putative	GL50803_15871		X	X	
Variant-Specific Surface Protein 88 (VSP-88)	GL50803_101074	X	X	X	
Leucine-rich repeat protein	GL50803_5795		X	X	
Alanyl dipeptidyl peptidase	GL50803_15574	X	X	X	
High cysteine membrane protein Group 1	GL50803_15317	X	X	X	
Bip (BiP)	GL50803_17121	X	X		X
Dipeptidyl-peptidase I precursor	GL50803_8741	X	X	X	
Peptidyl-prolyl cis-trans isomerase B precursor	GL50803_17000		X		X
Tenascin precursor	GL50803_8687		X	X	
Alpha-7.2 giardin	GL50803_114119		X		X
Variant-Specific Surface Protein 186 (VSP-186)	GL50803_14586	X	X	X	
Phospholipase B	GL50803_93548		X	X	
Neurogenic locus Notch protein precursor	GL50803_16322		X		X
5' nucleotidase family protein	GL50803_92645		X	X	
Lysosomal acid phosphatase precursor	GL50803_7556		X	X	
High cysteine membrane protein Group 2	GL50803_16721		X	X	
Variant-Specific Surface Protein 71 (VSP-71)	GL50803_137681		X	X	
Variant-Specific Surface Protein 53.1 (VSP-53.1)	GL50803_11470	X		X	

## Discussion

Considering the limited amount of data concerning *G*. *duodenalis* strains isolated from dogs, proteomic characterization, especially of those strains belonging to zoonotic assemblages, is an interesting approach, providing new tools to study parasite’s biology. Moreover, the discovery of novel proteins, enzymes and antigens is relevant for developing biotechnological strategies aiming to improve the diagnosis and prevention of giardiasis.

The consensus Baeysian phylogenetic analysis, based on three different gene sequences of *G*. *duodenalis*, showed that BHFC1 has a common ancestor with strains of the assemblage A, and is more closely related to sub-assemblage AI strains. The fact that the phylogeny based on *bg* gene clustered BHFC1 in sub-assemblage AII may be explained by recombination events in this locus. A similar hypothesis has been previously raised to explain discordant genotyping results and assemblage clustering obtained after sequencing genes of *G*. *duodenalis* isolates [24]. Thus, besides confirming our previous data [19], this analysis further refined BHFC1 characterization, indicating its phylogenetic relationship to the sub-assemblage AI.

*G*. *duodenalis* strains belonging to assemblage A do not always cause infection in mice, unless animals are treated with antibiotics that affect the intestinal microbiota [25]. In agreement, the Portland-1 strain was less infective to Swiss mice, since low numbers of cysts were recovered after infection. In contrast, the BHFC1 strain was able to infect mice, with a large number of trophozoites recovered from the intestine after infection. The fact that only cysts could be recovered from Portland-1 infection compared to trophozoites from BHFC1 infection may be attributed to a loss of virulence or to faster transformation from trophozoites to cysts in the Portland-1 strain. Indeed, it is known that axenic cultivation for prolonged periods may lead to the loss of virulence in protozoans [26]. Additionally, the virulence of *G*. *duodenalis* infection is attributed to the ability of trophozoites to persist and attach to the host gastrointestinal tract.

The proteomic study of the BHFC1 strain allowed the identification of molecules that suggest a high active metabolic profile, including proteins associated to carbon metabolism, cellular trafficking and endoplasmic reticulum-mediated endocytosis [27–29]. Some of these proteins have been associated to virulence in other pathogens, including *Candida albicans* [30], *Shigella flexneri* [31] and *Pseudomonas sp* [32]. Although the metabolism of cultured trophozoites cannot always be associated to virulence, a proteomic study also have previously correlated virulence with a high metabolic activity [8].

The search for conserved domains revealed the presence of a phospholipase B domain in one identified hypothetical protein. This is an interesting point, since *G*. *duodenalis* trophozoites have phospholipase A2 (PLA2) activity in sub-cellular fractions, but no phospholipase genes have been found in *G*. *duodenalis* genome [33]. Moreover, the PLA2 activity in sub-cellular fraction has been associated with virulence in some protozoans [34, 35]. Intriguingly, *G*. *duodenalis* is not able to synthesize phosphatidylethanolamine (PE) and phosphatidylglycerol (PG), two of the phospholipids present in trophozoites and cysts, and seems to generate these compounds by base-exchange reactions rather than *de novo* synthesis [36]. These phospholipids components may be obtained directly from the growth media or from degradation of complex molecules by phospholipase activity. Therefore, phospholipases may be an important element of phospholipids metabolism in *G*. *duodenalis*, and deserve further investigation on their function, structure and biological roles.

Twenty-seven identified proteins had been described only in the *G*. *duodenalis* genome therefore being hypothetical proteins. Thirteen of these proteins have conserved putative domains, though they all have high similarity to *G*. *duodenalis* and low similarity to other parasite’s molecules.

Five of the proteins found in this work presented homology to proteins of other parasites (S4 table). RhoGAP domain-containing protein (*Entamoeba histolytica* HM-1: IMSS) is part of the Rho GTPase proteins that have emerged as key players in the regulation of a variety of biological activities, including actin polymerization, adhesion, cell cycle progression and cell polarity, and are common in eukaryotes [37]. The role of RhoGAP domain proteins in *G*. *duodenalis* has not been characterized yet, although, according to the Smart^™^database, there are nine putative proteins in the genome of *G*. *duodenalis* presenting this domain [38]. Kinesins, here found related to *Trypanosoma cruzi*, have been described as an important virulence factor for this parasite. During *T*. *cruzi* invasion of the host cells, lysosomes are mobilized to the site of parasite attachment by a microtubule/kinesin-mediated transport. [39]. In *G*. *duodenalis*, only one study reported that kinesin-13 is responsible for regulating the flagella extension of *G*. *duodenalis* [40].

In conclusion, the proteomic analysis of the BHFC1 strain led to the identification of several proteins, including hypothetical proteins, which may be related to an active metabolic and virulent profile of this *G*. *duodenalis* canine strain. Further studies on the protein function, expression and structure of these newly identified proteins may reveal their role in *G*. *duodenalis* biology. Additionally, due to its infectivity to mice, BHFC1 becomes a valuable tool for a better understanding of *G*. *duodenalis* biology and more amenable laboratory model.

## Supporting Information

S1 Table Primers used for the nested PCR amplification of gene sequences used in phylogenetic analysis of BHFC1 strain. (XLSX)

S2 Table Gene sequences of groups and outgroups used for construction of phylogenetic trees. (XLSX)

S3 Table Proteins identified in the BHFC1 proteome. (XLS)

S4 Table Proteins identified in the proteomic map of BHFC1, which are related to other parasite proteins. (XLSX)
